# Swollen Breasts

**Published:** 1834

**Authors:** 


3. *Remedy for Swollen Breasts.*—Dr. Ranque, a continental physician, (*Dublin Journal*) has recommended for this common malady, the composition in the following extract:

"The swelling of the breast which precedes the formation of mammary abscess, is caused in the first instance by the retention of the milk and the consequent distention of the lactiferous ducts. But this is not the only cause of the local derangement that so speedily follows, for the vascular system of the mammæ is wonderfully increased preparatory to and during lactation, and, therefore, when this augmented circulation of the breasts is baffled in the performance of its proper function, the secretion of milk, it often tends to form with great rapidity vicarious and unhealthy products. Hence arises the obstinacy of many such cases, and hence they are frequently not found to be amenable to the common methods of treating local congestions or inflammations. All practical men are consequently obliged to adopt various methods of treatment, and the skilful accoucheur is often enabled by attention and pains to save his patient from the suffering accompanying such affections. Ranque, impressed with certain theoretical ideas which it is unnecessary here to discuss, was led to the use of the following liniment:—

> ℞. Extracti Belladonnæ, ℈ ii.

> Aquæ Laurocerasi, ℨ ii.

> Ætheris Sulphurici, ℨ i.

> Ft. Linimentum.

This must be well shaken before it is used. It is to be rubbed into the breasts as high as the axillæ, morning and evening, and the breast must be then covered with fine flannel soaked in the liniment. This proceeding must be repeated every day, until the swelling disappears, which is usually on the second or third day. The æther has a smell which to some is very disagreeable, but they ought to bear this inconvenience if possible, for it adds essentially to the efficacy of the remedy."

